# The genetic evidence for human origin of Jivaroan shrunken heads in collections from the Polish museums

**DOI:** 10.1007/s00414-016-1448-7

**Published:** 2016-09-18

**Authors:** Danuta Piniewska, Marek Sanak, Marta Wojtas, Nina Polanska

**Affiliations:** 1 Present address: Department of Forensic Medicine, Jagiellonian University Medical College, Grzegorzecka Str. 16, 31-531 Krakow, Poland; 2 Department of Internal Medicine, Jagiellonian University Medical College, Krakow, Poland

**Keywords:** *Tsantsa*/shrunken head, Short tandem repeats, Y-chromosome haplotype, Forensic identification

## Abstract

Advances in forensic identification using molecular genetics are helpful in resolving some historical mysteries. The aim of this study was to confirm the authenticity of shrunken-head artifacts exhibited by two Polish museums. Shrunken heads, known as *tsantsas*, were headhunting trophies of South American Indians (Jivaroan). A special preparation preserved their hair and facial appearance. However, it was quite common to offer counterfeit shrunken heads of sloths or monkeys to collectors of curiosities. We sampled small skin specimens of four shrunken-head skin from the museum collection from Warsaw and Krakow, Poland. Following genomic DNA isolation, highly polymorphic short tandem repeats were genotyped using a commercial chemistry and DNA sequencing analyzer. Haplogroups of human Y chromosome were identified. We obtained an informative genetic profile of genomic short tandem repeats from all the samples of shrunken heads. Moreover, amplification of amelogenin loci allowed for sex determination. All four studied shrunken heads were of human origin. In two ones, a shared Y-chromosome haplogroup Q characteristic for Indigenous Americans was detected. Another artifact was counterfeited because Y-chromosome haplogroup I2 was found, characteristic for the Southeastern European origin. Commercial genetic methods of identification can be applied successfully in studies on the origin and authenticity of some unusual collection items.

## Introduction

The Jivaroan are known as Amazonian groups of indigenous peoples living in the headwaters of the Marañon River and its tributaries, in the mountainous region of northern Peru and eastern Ecuador. They are the second largest and one of the most studied Amazonian groups with a notable history of survival and defense against outsiders. The Jivaroan represent a small linguistic family consisting of dialects: Shuar, Achuar, Aguaruna, and Huambisa. All of them share similar customs and they are genetically related [1, 2].

In the nineteenth century, the Jivaroan peoples became famous among Europeans and Euro-Americans travelers for their elaborate process of taking and shrinking an enemy’s head to make a *tsantsa*. Although headhunting has occurred in many regions of the world, the practice of shrinking human heads has only been documented in the northwestern region of the Amazon rainforest, performed by the Jivaroan peoples. The reason for that had a spiritual origin rather than to keep them as a trophy. According to Jivaroan beliefs, a vengeful soul or *muisak* of a victim had to be trapped inside the *tsantsa* to protect the killer against the revenge. This practice also prevented the *muisak* from entering the afterlife potentially harmful for the family of the murderer or could be useful to enhance yields of crops [1, 2].

*Tsantsas* were made from men, women, and children. Steps in the process of shrinking the heads had often to be carried in camps along the way back home after the attack. The head was severed by cutting the skin at the extreme base of the neck, in a “V” shape just above the clavicles. The skull was often submerged in a river to ease separation of muscles and tendons connecting the skin to the skull. The skin was severed from bones using a knife and eyes were removed. The skin of the head with the hair attached was sunk into the boiling water for a period between 30 min to 2 h three times and then placed on a spear to dry. Boiling led to a significant contraction of the skin to about one third of the original size. Then, the mouth and eyelids were sewn shut and nostrils were sometimes plugged with a cotton or a pitch. Next, hot small pebbles and sand were filled into the interior of head to complete the making of the shrunken head. Ultimately, the head was dangled over a fire to darken and toughen the skin. Alternatively a charcoal ash was rubbed over the skin and a hot blade was pressed to the lips to dry them. After these steps generally taking from 2 to 3 days, a human head was reduced in size to about one fourth, roughly to the size of a fist. During this process, the hair was not reduced, lending a characteristic look of the small head with long hair [1–3]. The spiritual system underlying the manner in which these trophies were made and celebrated changed during the second half of the nineteenth century when the Europeans arrived in the area. *Tsantsas* became used as a currency of trade. This created an economic demand for shrunken heads and superseded the spiritual and practical reasons for headhunting. The rate of killings rose in order to supply collectors and tourists with *tsantsas*, which resulted in many tensions within the Jivaro world [4]. Nowadays, shrinking-of-heads practices are prohibited.

Requests for shrunken heads also encouraged other people, non-Jivaroan, to produce counterfeited *tsantsas* for purposes other than those originally intended. The heads were usually taken from sloths and monkeys or even from human morgue corpses [4, 5]. After the World War II, two shrunken heads were found at the Buchenwald concentration camp and were among evidences in Nuremberg Nazi Trials because they were thought to be taken form prisoners [6]. Even the Jivaroan peoples sometimes made a *tsantsa* of a sloth’s head as they believed that it was the only animal in the forest that had a vengeful soul. It served as a remedy for a warrior who successfully killed the enemy but failed to take his head. Sometimes, it was also a part of a passage ceremony by boys to enter their manhood [3].

Nowadays, it is estimated that up to 80 % of shrunken heads found in museums or in private collections are counterfeits. Very few fake *tsantsas* were ever made closely following the Jivaroan methods [7]. An audit conducted in the 1990s at the Smithsonian’s collection revealed that only five of 21 examined were authentic *tsantsas* made by the Jivaroan [8]. Previous studies on the shrunken heads were based on the macroscopic examination of the head and microscopic analysis of hairs and skin to determine their origin [3, 4, 9–11]. This examination was usually sufficient for confirmation of authenticity because forgers did not concern about traditional Jivaroan methods of preparation. They wanted the head to look as lifelike as possible to please the buyers. Despite this, some counterfeiters could have produced *tsantsas* almost identical to those made by the Jivaroan peoples and anatomy or microscopic analysis could fail [4]. Molecular genetics used in forensic medicine offer an advantage of authentication by DNA analysis, which is increasingly used by museums or private collectors [5, 10, 12]. The authenticity certificates are sometimes important because false or non-ceremonial heads do not have to be a subject of eventual repatriation requested upon museum curators or private owners [13, 14]. The National Museum of the American Indian at the Smithsonian was the first one to repatriate *tsantsas* to the Shuar Federation in 1999 [15].

**Fig. 1:**
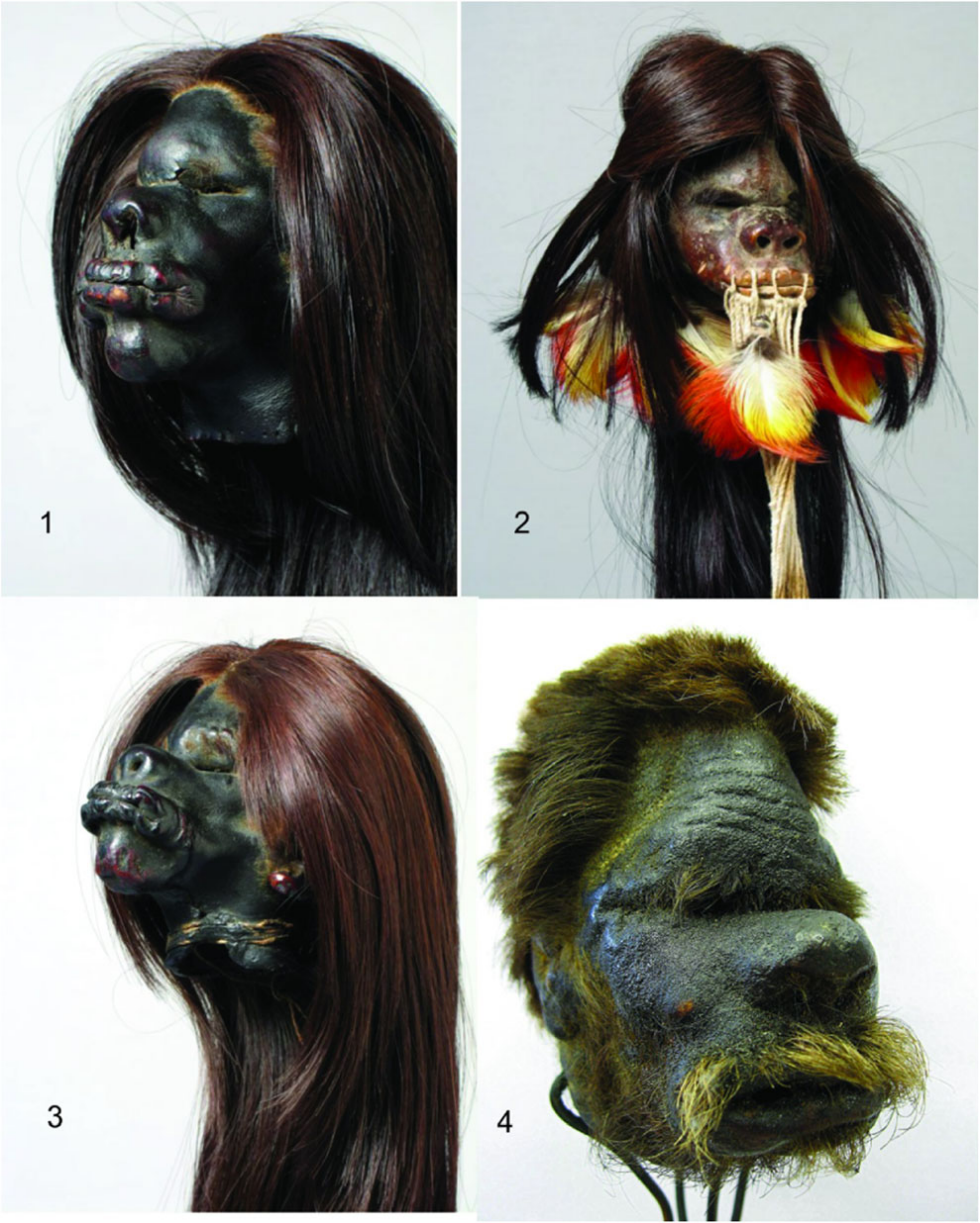
View of the shrunken heads (The State Ethnographic Museum, Warsaw, Poland: *no.1- PME 5261*, *no.2- PME 12272*, *no.3- PME 5260*; The Museum of the Department of Forensic Medicine at Jagiellonian University Medical College, Krakow, Poland: *no.4*)

The aim of the current study was to confirm a human origin of the three shrunken heads preserved in the State Ethnographic Museum in Warsaw (no.1- *PME 5261*, no.2- *PME 12272*, no.3- *PME 5260*) and one in the collection of the Museum of the Department of Forensic Medicine at Jagiellonian University Medical College in Krakow (no.4), Poland (Fig. 1).

The *tsantsa* from the State Ethnographic Museum in Warsaw originated from Ecuador (no.3*- PME 5260* and no.1*- PME 5261*) and from Peru (no.2- *PME 12272*). The first two were purchased from Mr. Vladimir Orda in 1950. The third was purchased by Mr. Stanislaw Jamka, who acquired it from the Indians in exchange for the rifle and beads in the year 1934. The first notice about the exhibit no.4 in the inventory of the Department of Forensic Medicine appeared in the year 1903. It is known that experiments on *tsantsa* preparation were conducted at the local autopsy room in the 1970–1980s of the nineteenth century, when Ludwig Teichmann was the head of the Department of Forensic Medicine in Krakow. This shrunken-head hair was cut to make them match the head size.

## Material and methods

Genetic analysis was carried out in the DNA laboratory certified for forensic genetics and with respect to the all precautions to avoid contamination with other human DNA. Briefly, laboratory steps of identification were performed in separate rooms using laminar-flow hoods, sterile disposable plasticware, and reagents. Pipettor tips with filters were used for all liquid handling. Negative PCR controls of deionized water were used throughout the whole process.

The external layer of the skin was cleaned several times with 99.8 % ethanol and flat cut using disposable and sterile scalpel blade. Then, samples (∼0.5 cm^2^ and 0.1 cm thick) were taken from the inner surface of the neck in all four shrunken heads. This surface had no direct contact with the outside environment. Each sample was collected into a sterile 1.5-mL Eppendorf tube and incubated overnight at 42 °C prior to DNA extraction. DNA was extracted by enzymatic digestion and ion-exchange column extraction using *Sherlock AX* kit (A&A Biotechnology, Gdynia, Poland) according to the manufacturer’s recommendations. The concentration of DNA was determined by spectrophotometry at 260 nm using a GeneQuant RNA/DNA Calculator (Pharmacia Biotech, Cambridge, UK). PCR amplification was performed in a standard thermocycler (Perkin Elmer GeneAmp 2400, Applied Biosystems, Foster City, USA) and contained 4 ng template DNA in a final reaction volume of 25 μL. Five commercially available reagent kits for typing of genomic short tandem repeat (STR) markers were used to study nuclear DNA (nDNA): AmpFlSTR Identifiler PCR Amplification Kit, GlobalFiler Amplification Kit, AmpFlSTR YFiler PCR Amplification Kit, and AmpFlSTR YFiler Plus PCR Amplification Kit (all from Applied Biosystems, Foster City, USA). Investigator Argus X-12 Kit was used to type for X-linked STR (Qiagen GmbH, Hilden, Germany). These reagents were validated by the manufacturer and had appropriate genotypic controls and markers allelic ladders. The amplification products were analyzed using the AB3500 Genetic Analyser (Applied Biosystems, Foster City, USA). Fragment size and allele designation were determined by comparison with allelic ladders. The analytical threshold used for peak detection was set on 100 RFU due to the high degradation of genetic material. Results were analyzed using GeneMapper ID-X Software (Applied Biosystems, Foster City, USA).

Minisequencing was performed for the Y-Chromosomal SNP marker M3 (rs38940) and M438 (rs17307294) in the male samples. Primer sequences used for amplification and sequencing were as follows: M3F-GGTACATTCGCGGGATAA; M3R-GAATCTGAAATTTAAGGGCATC and M438F-GTGGCTGCATAAGGTAGATAAT; M438R-AATTCTCTGATGGCGAGTC. Products of amplification were separated on agarose gel (M3—144 bp; M438—237 bp) and extracted, next sequenced using BigDye Terminator v3.1 Cycle Sequencing Kit according to the manufacturer protocol and AB 3500 Genetic Analyser. Sequence analysis was performed with Sequencing Analysis Software v3.7 (Applied Biosystems, Foster City, USA).

Comparisons of Y-STR haplotypes were carried out against YHRD database release 51 (Y-Chromosome Haplotype Reference Database; available at http://www.yhrd.org). Haplogroup predictor software was used for a haplogroup ascertainment (www.hprg.com/hapest5/). The family relationship between tentatively consanguineous samples (no.1, no.2, no.3) was tested using DNA-View software v. 33.17 (Brenner C, Iowa City, USA).

Hair was collected from the tested exhibits by pulling them out with tweezers and inspected under a light microscope (Zeiss, Axioscope; Jena, Germany).

## Results

The examined exhibits were genotyped for 21 autosomal STR markers (Table 1). Each of the tested samples revealed an incomplete autosomal STR profile. Failures to genotype were more common for the reaction products which size exceeded 250 bp. Amelogenin amplification products determined human sex in all four samples. Three of four *tsantsas* were males and one was a female. No amplification products have been ever observed in multiple blank extraction and PCR controls. The use of two different kits for the amplification of autosomal (Identifiler/GlobaFiler) and Y-STRs (Yfiler/Yfiler Plus) markers allowed to crosscheck the reliability of the results. Autosomal alleles almost completely overlapped for the both kits, whereas in two markers (TPOX, D18S51), better results were obtained using Identifiler. Analysis of Y-STR profile showed better amplification using Yfiler Plus kit. The genetic analysis showed progressive degradation a template quality with the amplicon size in all tested samples. The peak heights decreased with their migration time in the electropherogram and some allelic or locus dropouts were noticed.

**Table 1:** The genetic profiles of the shrunken heads. Autosomal STR and X or Y amelogenin alleles

STR loci	Shrunken head no.1	Shrunken head no.2	Shrunken head no.3	Shrunken head no.4
D3S1358	15	17	15–16	17–18
vWA	16	15–19	14	15–19
D16S539	10–11	10	10–12	–
CSF1PO	–	10–12	12	–
TPOX	8–11	11–12	12	8
Y indel	2	–	2	2
Amelogenin	XY	X	XY	XY
D8S1179	11–14	10–12	11–13	13–15
D21S11	31.2–33.2	31.2	29–33.2	30.2–32.2
D18S51	14–18	14–15	14–15	13–14
DYS391	–	–	10	–
D2S441	10–11	10–11	11–16	10–11
D19S433	13–15	14.2	14.2–15	13.2–14
TH01	7–9.3	6–7	7–9.3	6–8
FGA	22–25	21–24	24–25	20–21
D22S1045	15–16	16	15–16	11–16
D5S818	11	9–11	11–13	9–12
D13S317	9	9–10	9	12–13
D7S820	12–14	10–11	10	–
SE33	17–26.2	18–27.2	26.2–29.2	–
D10S1248	14	14 – 15	9–14	13–16
D1S1656	15–17.3	13–17	14–15	15
D12S391	19–20	19	18–19	17.3–18
D2S1338	18–20	18–20	17–20	–

The results of autosomal STR profiles from investigated *tsantsa* were compared to the only available profile obtained from the shrunken head displayed at the “Eretz Israel Museums, Tel-Aviv” and were found to be different [12].

The Y-chromosomal haplotype of the three male shrunken heads was obtained for 25 Y-STR markers (Table 2). Only the DYS533 marker did not reveal alleles for two samples (no.1, no.4). Interestingly, the two of the three individuals shared identical haplotype (no.1, no.3). In a search for geographic origin of the artifacts, we compared Y-STR haplotypes with published data on ethnic groups from Ecuador [16–19]. None of the obtained haplotypes had been previously observed among inhabitants of Ecuador. However, the modern ethnic composition in Ecuador is rather admixed. Mestizos are the largest population group (mixed descendant of indigenous Amerindians and Spanish colonists), who constitute just over 65 %; others are Indians (25 %), Europeans (7 %), and Afro-Ecuadorians (3 %) [16]. Noteworthy, the Y haplotype of the shrunken heads differed from the Waorani, the last semi-nomadic population of hunter-gatherer horticulturalists living in the Amazon region of Ecuador [17, 20].

**Table 2:** The genetic profiles of the shrunken heads. Y-chromosomal STR alleles

Y-STR loci	Shrunken head no.1	Shrunken head no.3	Shrunken head no.4
DYS576	18	18	17
DYS389I	14	14	12
DYS635	22	22	21
DYS389II	32	32	30
DYS627	20	20	19
DYS460	10	10	10
DYS458	19	19	17
DYS19	12	12	16
YGATAH4	11	11	11
DYS448	20	20	19
DYS391	10	10	10
DYS456	17	17	15
DYS390	23	23	24
DYS438	11	11	10
DYS392	14	14	11
DYS518	41	41	40
DYS570	18	18	18
DYS437	14	14	15
DYS385	14**–**17	14**–**17	14**–**15
DYS449	31	31	29
DYS393	14	14	13
DYS439	12	12	12
DYS481	24	24	30
DYF387S1	35**–**41	35**–**41	37**–**39
DYS533	**–**	11	**–**

The Y-Chromosome Haplotype Reference Database (YHRD; http://www.yhrd.org), was searched to answer whether two different haplotypes found in the examined shrunken heads existed in other populations. We found no entry among 6 872 Y-STR haplotypes reported in this database. We also searched the most frequent minimal haplotypes within the YHRD database (i.e., *DYS19-389I-389II-390-391-392-393-385*). One match in 160,693 worldwide reported haplotypes was found for sample no.1 and no.3 in Ecuador population, whereas five matches were observed for sample no.4, including two hits in the Eurasian-European-Eastern European populations (Hungary, Slovakia, Macedonia).

Using comparisons provided by Haplogroup Predictor (http://www.hprg.com/hapest5/) based on 22 Y-STR markers, 100 % probability of Q haplogroup was obtained for sample no.1 and no.3. These findings were reinforced by previous observations that all Waorani males belonged to haplogroup Q. This is the major lineage among the Native Americans, with Q-M3 (Q1a3a) being almost completely restricted to the Americas [20, 21]. A recent publication on the Waorani stated that more than 90 % of the males belonged to a sub-haplogroup Q1a3a [22]. For the sample no.4, probability of I2a haplogroup was also 100 %. The haplogroup I represents one of two major European Y chromosome haplogroups [23–25] and by origin is absent elsewhere. Subclade I1 is the most frequent in the Northern Europe, whereas subclade I2 is the most frequent haplogroup in the Eastern Europe and the Balkans [24]. This coincides with the origin of the head and the reported history of its preparation. A contemporary contamination of this exhibit with a single individual DNA could not be excluded. However, a similar pattern of nuclear DNA template degradation in all samples and no traces of any other profile admixture advocates against pre-laboratory contamination.

Additionally, in order to confirm the origin of the *tsansta* haplotype, minisequencing was performed for the Y-Chromosomal SNP markers: M3 for Q1a3a haplogroup and M438 for I2 haplogroup. These results confirmed the predicted haplogroup Q1a3a of native South Americans for *tsantsa* no.1 and no.3 (rs3894C > T variant).

**Fig. 2:**
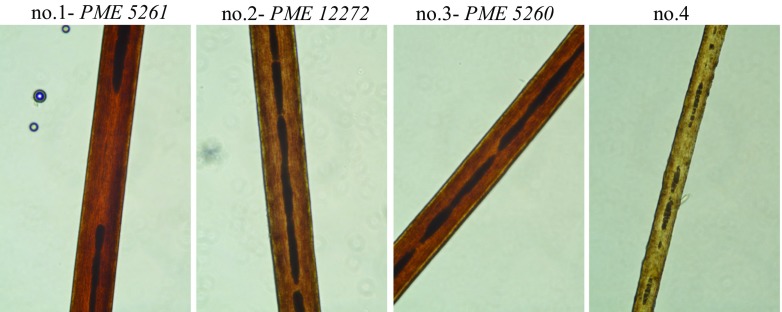
Microscopic view of hair samples from the shrunken heads (images at ×40 magnification)

We also investigated X-chromosome markers. Out of the four shrunken heads, only three were successfully typed for 12 X-STR’s markers (Table 3), all from the State Ethnographic Museum in Warsaw (no.1, no.2, no.3). The samples no.1 and no.2 revealed a complete X-STR profile; a partial one was obtained for the sample no.3. Genotyping of the sample no.4 ascertained partial genotype of four X-STR markers, and this failure could result from partial degradation of DNA stored frozen for a few months before the assay. All the samples had different X-STR profiles and the subjects were not closely related through the maternal line. We compared these data to the ethnic groups from Ecuador [20, 26, 27]. None of the resulting profiles had been previously observed in these populations.

**Table 3:** The genetic profiles of the shrunken heads. X-chromosomal STR alleles

X-STR loci	Shrunken head no.1	Shrunken head no.2	Shrunken head no.3	Shrunken head no.4
AMEL	XY	XX	XY	XY
DXS10148	24.1	27.1	26.1	–
DXS10135	15	24	20	–
DXS8378	10	10	10	9
DXS7132	14	13–15	–	–
DXS10079	22	20–25	–	–
DXS10074	15	15–18	18	8
DXS10103	19	16	19	20
HPRTB	14	13–15	14	–
DXS10101	30.2	33	30.2	–
DXS10146	28	25–28	27	27
DXS10134	38	39	34	–
DXS7423	15	14–15	17	–

To estimate the probability of kinship between the *tsantsa* from the State Ethnographic Museum, especially heads no.1 and no.3, we performed statistical calculations based on the autosomal markers using automatic kinship module of DNA-View software. There was no evidence for familial relationships between the individuals no.1, no.2, and no.3, based on the population of Amerindian Kichwas [28], Ecuador [29], and Peru [30]. The probabilities were less than 0.3 (LR = 0.03) for the hypothesis that the heads no.2 and no.3 are biological siblings. Even lower values were obtained for the heads no.1 and no.3. A hypothesis that they were half-siblings with a common father was not confirmed (LR = 1.074). However, using Brazilian Amazon Region population frequencies [31], individuals no.1 and no.3 had 0.99053 probability (LR = 104.6) of having a common father/male ancestor, which could explain their identical Y-STR profile. Assuming that they were biological siblings, probability was 0.8590 (LR = 6.092) using the Brazilian Amazon Region data while for the remaining population data LR value dropped below 0.5.

Hair samples from tested exhibits studied under microscope (Fig. 2) had no hair roots visible. Apparently, this was not possible to obtain the roots while collecting samples from the hard and shrunk scalp. It is also plausible that the process of preparation (boiling in water and drying over fire) could destruct hair roots. The hair medulla was less than one-third width of the shaft, amorphous, and mostly discontinuous (Fig. 2). These observations were compatible with human hair histology.

## Conclusions

Analysis of nuclear short tandem repeats located at autosomal or sex chromosomes proved that all the studied shrunken heads were of human origin. Nevertheless, Y-STR haplogroup I2 of the sample no.4 suggested a Southeastern European ancestry precluding a genuine Jivaroan origin. Two other samples (no.1, no.3) were Amerindians and probably consanguineous by a common male ancestor, because they shared identical profile of Y-chromosome haplogroup Q1a2-M3. This haplogroup is characteristic for the Native Americans (Ecuador).

Calculations using automatic kinship module of DNA-View software did not provide any strong evidence of familial relationships between the individuals no.1 or no.2 and no.3 using autosomal STR but it was plausible that individuals no.1 and no.3 had likely (LR = 104.6) a common male ancestor. They also shared identical Y-STR haplotype.

Certification of *tsantsa* authenticity by macroscopic or microscopic analyses sometimes is disputable. Preparation of some counterfeiters may employ a process very similar to the genuine *tsantsa* preparation by the Jivaroan peoples. On the other hand, some variants of the classic technique among authentic *tsantsas* also existed, or alternatively, some later modifications could have been done by a later owner. The genetic profiling together with traditional anthropological identification techniques seemed very helpful for determining the human origin of a shrunken head and their geographic provenance.
